# The impact of environment-friendly short videos on consumers’ low-carbon tourism behavioral intention: A communicative ecology theory perspective

**DOI:** 10.3389/fpsyg.2023.1137716

**Published:** 2023-02-09

**Authors:** Xin Chen, Zhen-feng Cheng

**Affiliations:** ^1^College of Landscape Architecture and Tourism, Hebei Agricultural University, Baoding, China; ^2^Business School, Guangzhou College of Technology and Business, Guangzhou, China

**Keywords:** communicative ecology theory, environment-friendly short video, low-carbon tourism behavioral intention, empathy with nature, perceived environmental responsibility

## Abstract

As key participants in tourism activities, the tourists have an important role in the carbon emissions. Therefore, it is essential to identify the key elements that can arouse consumers’ low-carbon tourism behavioral intention; this has become an important topic for the academic community. However, to the best of my knowledge, most studies have explored the process of formation of consumers’ low-carbon tourism behavioral intention from the cognitive or emotional perspective, and have seldom paid attention to the communication perspective. As a result, the interpretation and prediction of consumers’ low-carbon tourism behavioral intention is limited. Based on the framework of communicative ecology theory (CET) and stimulus-organism-response theory (SOR), our study constructs an integrated model of the relationship between environment-friendly short video experience and consumers’ low-carbon tourism behavioral intention at the technological, content and social levels, introduces emotional elements such as empathy with nature and perceived environmental responsibility. Structural equation model and bootstrap method were employed for analyzing the data. Results revealed that the presence and perception of environmental education are the cognitive factors that impact consumers’ low-carbon tourism behavioral intention; these can effectively stimulate consumers’ low-carbon tourism behavioral intention. Empathy with nature and perceived environmental responsibility are the emotional factors that impact consumers’ low-carbon tourism behavior; they play significant mediating roles between environment-friendly short video experience (presence, perception of environmental education, online interaction) and consumers’ low-carbon tourism behavioral intention. On the one hand, the research conclusions enrich the perspective and content of the research on consumers’ low-carbon tourism behavioral intention and its influencing mechanism; on the other hand, they acquaint with the practical significance of implementing environmental education *via* the emerging communication modes such as short videos, improve consumers’ awareness of their environmental responsibility, and promote environmental governance and sustainable development of tourist destinations.

## 1. Introduction

Currently, global warming has become an issue of great concern for the international community, which is posing a serious threat to the environment and natural ecology on which human beings depend for survival (Dai et al., 2022). In this context, the carbon emission due to tourism has aroused widespread concern. According to the research report of the World Tourism Organization and United Nations Environment Programme, the carbon emissions due to tourism account for 4.9% of the total carbon emissions due to human activities, and the greenhouse effect that results from the carbon emissions from tourism accounts for about 14% of the global effect. This not only causes further increase in global warming, but also threatens the sustainable development of the tourism industry itself (Sung et al., 2021). Therefore, exploring the low-carbon tourism development mode is an important problem that needs to be solved urgently by the tourism academia and industry.

As the main participants in tourism activities, tourists have an important impact on the carbon emissions of tourism (Horng and Liaw, 2017). Some studies have pointed out that the key to realizing low-carbon tourism is to promote change in behavior of individual tourists, which can directly reduce energy consumption and carbon emissions (Fakfare and Wattanacharoensil, 2022). Therefore, effectively identifying the key elements that can activate consumers’ low-carbon tourism behavioral intention has become an important issue for the academic community. Existing studies on the antecedent variables of tourists’ environmentally responsible behavior mostly focus on tourists’ cognitive perspective (environmental knowledge, tourist-environment fit, etc.; Wang and Zhang, 2020; Cai and Zhu, 2021) or emotional perspective (green commitment, low carbon attitude, place attachment; Hu et al., 2014; Cai and Zhu, 2021; Shie et al., 2022). The influencing mechanism of low-carbon tourism behavioral intention has rarely been explored from the perspective of communication.

In recent years, digital technology has permeated into and benefited all aspects of industrial systems and daily life. With the development of new media technology, the sources of communication are no longer confined to words and pictures, but short videos have been rapidly developed and popularized, that have greatly improved the communication efficiency (Dong and Tian, 2018). Diversified forms of short videos can attract users’ attention more effectively, promote knowledge transmission, and thus serve as an important channel for information transmission (Cao D. M. et al., 2021). In this context, the government and various environmental protection organizations have launched a large number of environment-friendly short videos in an attempt to strengthen the public’ s understanding of ecological knowledge and promote practice of low-carbon tourism behaviors. What is the actual effect of such environment-friendly short videos? Does it strengthen public awareness of environmental protection? Does it effectively stimulate consumers’ low-carbon tourism behavioral intention? To the best of my knowledge, there is lack of empirical evidence for such relevant issues, which need urgent solution through systematic exploration by the academic community.

However, existing studies on the impact of short videos mainly focus on destination image publicity and formulation of marketing strategies (Ke et al., 2022; Zhao et al., 2022), and less attention is paid to the relationship between short videos and consumers’ low-carbon tourism behavioral intention. Can environment-friendly short videos effectively enhance consumers’ willingness to indulge in low-carbon tourism behavior? These relevant issues are yet to be clarified. In addition, few studies have explored the mediating mechanism in the formation of consumers’ low-carbon tourism behavioral intention. Hence, our study tries to understand the impact of environment-friendly short videos from the perspective of emotional response, explore the mediating role of empathy with nature and perceived environmental responsibility between short videos and consumers’ low-carbon tourism behavioral intention. Communicative ecology theory (CET) lays a theoretical foundation for analyzing the influencing mechanism of environment-friendly short videos on the consumers’ low-carbon tourism behavioral intention. According to CET, the media communication effect is the result of the comprehensive effect of content, social and technological factors (Altheide, 1994; Foth and Hearn, 2007). Since the environment-friendly short video platform integrates technology, environmental knowledge and online interaction, it is necessary to systematically explore its communication effect from the perspectives of technology, content and social interaction.

In view of this, taking the communicative ecology theory (CET; Altheide, 1994) and the stimulus-organism-response theory (SOR; Mehrabian and Russell, 1974) as the theoretical basis of the study, a mediation model based on empathy with nature and perceived environmental responsibility is established via our study which systematically explores the specific path of the effect of environment-friendly short video experience on consumers’ low-carbon tourism behavioral intention. The main objectives of our study are as follows: (1) To explore the influencing mechanism of presence (technological layer), perception of environmental education(content layer) and online interaction (social layer) on consumers’ low-carbon tourism behavioral intention, and to pursue in-depth research to explore the formation mechanism of consumers’ low-carbon tourism behavioral intention; (2) To clarify the mediating mechanism of empathy with nature and perceived environmental responsibility between environment-friendly short video experience and consumers’ low-carbon tourism behavioral intention; (3)To provide a new research perspective for revealing the influencing path between environment-friendly short video experiences and consumers’ low-carbon tourism behavioral intention; (4) To extend the CET to the field of tourism research and expand the application range of relevant theories.

## 2. Theoretical review and research hypotheses

### 2.1. Communicative ecology theory

Communicative ecology theory (CET) was first proposed by Altheide (1994) which emphasizes on understanding the relationship between communication media and the surrounding environment from a holistic perspective (Foth and Hearn, 2007). Altheide (1994) proposed the concept of communicative ecology on the basis of media ecology, with the aim to explore the interaction between information technology, communication paradigm and social activities in physical and digital environments. Foth and Hearn (2007) explored CET in depth in their research and divided CET into three layers: technological layer, content layer and social layer. The technological layer includes the technical system and communication media for interaction. The content layer (also known as discursive layer) focuses on the actual content of communication. The social layer consists of the different people involved and their social relationships. The three levels of CET provide a clearer picture for the study on the impact of social media (Seol et al., 2016). Review of literature revealed that existing researches on media communication have mainly focused on technological characteristics or content dimensions of media for exploring its communication effect (Kwon and Wen, 2010; Kim et al., 2015), and have rarely incorporated social dimensions into theoretical models. Therefore, our study introduced the CET, incorporated the social dimension into the analysis framework, and identified the influencing path of environment-friendly short videos on consumers’ low-carbon tourism behavioral intention in a more systematic way. As an important social media in daily life, short videos play an important role in interpersonal communication (Zhao et al., 2022). Therefore, the communication effect of short videos can be explored through the three layers of CET. Information technology devices are included in the short video technology layer, that allow people to connect and interact with each other; The content that people communicate on the platform constitute the components of the content layer of short video; the social layer of short video consists of the people involved and their interaction. According to CET, while exploring the influence of short videos on consumers’ behavioral intention, it is necessary to comprehensively explore the mechanism of technological layer (presence), content layer (perception of environmental education) and social layer (online interaction) of environment-friendly short videos on consumers’ low-carbon tourism behavioral intention from the integration perspective.

### 2.2. Presence (technological layer) and consumers’ low-carbon tourism behavioral intention

The concept of presence, derived from “telepresence” in communication, refers to an individual’s sensory experience of media environment (Lombard and Ditton, 1997), which generally includes two dimensions of spatial presence and social presence. Spatial presence refers to the degree of reality that an individual feels in a virtual environment, which reflects a kind of “immersive” feeling. When the perception experience of the virtual environment exceeds that of the real environment, spatial presence is generated. Social presence refers to the degree of interaction between individuals through communication media, reflecting a feeling of “being together with others” (Ou et al., 2014). In our study, presence mainly refers to the “immersive” feeling generated by individuals when browsing short videos, so it is included in spatial presence. The research on presence was first initiated in the fields of communication, human-computer interaction, distance education, etc. Later, it was widely used in the research of media environment because it can help us understand the perceptual process of individuals in media environment (Orth et al., 2019). Presence also exists widely in tourism situations and is crucial to understanding tourist behavior (Yao and Jia, 2021). Some scholars have pointed out that when individuals watch short videos, they are likely to be affected by the technical characteristics of the short videos, and the sense of presence has a positive promotional effect on individuals’ travel intention (Yung et al., 2021). In addition, when individuals watch live streaming pertaining to travel, a sense of presence is generated, and the stronger the sense of presence experience, the stronger the individual’s purchase intention for tourism products (Xu X. et al., 2021). In general, the existing literature reveals that presence plays a very important role in predicting the attitude and behavior of tourists, but the specific tourism scenarios are not included in the analysis framework. In view of the differences in people’ s psychological and behavioral patterns in different tourism contexts, it is necessary to focus on specific tourism contexts to deeply explore the impact of presence on tourists’ behavioral intentions. Therefore, this study focuses on low-carbon tourism scenarios and empirically explores the role of presence in predicting consumers’ low-carbon tourism behavioral intention.

Behavioral intention refers to the tendency of an individual to indulge in a specific behavior and is the antecedent of actual behavior (Harrison et al., 1997). In view of the important role that behavioral intention plays in predicting actual behavior, tourism scholars have been devoted to exploring the driving mechanism of tourist behavioral intention. Some scholars have conducted a large number of studies on the antecedent influencing variables of tourists’ behavioral intention from different perspectives, including the destination management and service aspects (tourism experience, service quality, etc.; Akroush et al., 2016; Chen et al., 2020; Cheng and Chen, 2022), and the tourists’ cognition and emotion aspects (emotion, place attachment; Hu et al., 2022; Zheng et al., 2022). Literature review shows that few studies have explored the influence of media communication on tourists’ behavioral intention based on the communication perspective. In recent years, with the development of digital technology and the popularity of intelligent terminals, short video has an increasingly significant impact on people’s life and work. Therefore, it is necessary to incorporate it into the framework of tourists’ behavioral intention analysis and systematically explore its important impact on tourist behavioral intention. In addition, with the deterioration of the global environment, it is urgent to explore the factors that can stimulate people’s willingness to engage in low-carbon tourism behavior (Fakfare and Wattanacharoensil, 2022). Therefore, our study focuses on low-carbon tourism context and empirically explores the mechanism of environment-friendly short video experience to promote consumers’ low-carbon tourism behavioral intention.

In recent years, the academic community has begun to pay attention to the relationship between presence and individual behavioral intention from the perspective of virtual reality and digital technology. Ye et al. (2020) provided evidence that presence in online websites can enhance customers’ intention to purchase online peer-to-peer accommodation. Yao and Jia (2021), through empirical analysis concluded that higher the degree of presence of online tourism information experienced by users, stronger is the impulse generated. Some scholars also explored the influence of presence on consumers’ behavioral intention based on online shopping scenarios. For example, Xu X. Y. et al. (2021) pointed out that presence of network broadcast has a significant positive impact on consumers’ purchase intention. Amin et al. (2021) found that the increase of presence can effectively promote consumers’ willingness to book hotels online. Ying et al. (2021) believed that higher the sense of presence in destination Virtual Reality (VR) experience, the stronger is the tourist intention to travel. Based on this, it was speculated that the higher the presence experienced by individuals while watching environment-friendly short videos, the more likely they are to have the tendency to engage in low-carbon tourism. Therefore, the following hypothesis was proposed:

*H1*: Presence has a significant positive impact on consumers’ low-carbon tourism behavioral intention.

### 2.3. Perception of environmental education (content layer) and consumers’ low-carbon tourism behavioral intention

Tourists’ perception of environmental education refers to their comprehensive perception of interventions pertaining to environmental education such as tour guides, destination managers, environmental interpretation boards and multimedia display systems (Li, 2012). The enhancement of tourists’ perception of environmental education can effectively improve the operation effect of the environmental education system, and achieve environmental education goals such as improving tourists’ environmental behavior, acquiring environmental knowledge and paying attention to environmental problems (Yu et al., 2022). From the perspective of educational objectives, perception of environmental education can be divided into three levels: the low level objectives seek to improve the environmental behavior of tourists in the destinations through educational intervention; the middle level seek to focus tourists’ attention to environmental issues through media publicity; and the high level focus on changing tourists’ attitude through inculcation of environmental values (Zhang et al., 2010). Existing studies on perception of environmental education and tourists’ behavioral intention are mostly carried out based on field tourism scenarios (Li et al., 2019), and little attention is paid to the relationship between ecological environment knowledge conveyed by short videos and tourists’ environmental responsibility behavioral intention. Therefore, our study empirically explores the impact of environmental education perception generated by watching short videos on consumers’ low-carbon tourism behavioral intention.

Relevant studies have pointed out that environmental education for tourists is one of the most effective ways to stimulate tourists’ pro-environment behavior (Li et al., 2019). There is a high correlation between tourists’ perception of environmental education and their intention to participate in environmental protection (Wang et al., 2022a), which can promote tourists’ pro-environment behaviors to a certain extent. On the contrary, lack of environmental education perception is likely to decrease tourists’ intention to protect the environment (Li et al., 2019). Positive low-carbon environmental education experience can facilitate increase in tourists’ environmental knowledge, enhance their cognition of low-carbon tourism, and cultivate an attitude of environmental protection in the tourists (Zhang et al., 2017a). In the empirical study on the environmental education system of eco-tourism destinations, Lee et al. (2022) found that interpretation services not only transmitted environmental knowledge, but also stimulated pro-environment behavior in the tourists. The study of Yu et al. (2022) also shows that environmental education for tourists in forest parks is conducive to the sustainable development of forest tourism. Tang et al. (2022) pointed out that tourists’ environmental behavior can be changed by participating in environmental educational activities, and environmental interpretation can effectively promote tourists’ responsible environmental behavior. Zhang et al. (2017b) also believes that perception of environmental education can improve tourists’ awareness of low-carbon tourism and stimulate low-carbon tourism behavior. Based on the above discussion, our study speculated that providing environmental education to consumers through short videos can facilitate development of their low-carbon tourism behavioral intention. Therefore, the following hypothesis was proposed:

*H2*: Perception of environmental education has a significant positive impact on consumers’ low-carbon tourism behavioral intention.

### 2.4. Online interaction (social layer) and consumers’ low-carbon tourism behavioral intention

As for the definition of online interaction, different scholars have provided different explanations from different perspectives. From the perspective of perception, Wu (2006) defines online interaction as users’ perception of the communication mode and their psychological feeling pertaining to the degree of control during the interaction. From the perspective of process, Tremayne (2005) believes that interaction is a process of information exchange, a process of mutual communication between interacting parties. From the perspective of resource exchange, online interaction is a process in which users exchange resources in network communities (Brack and Benkenstein, 2012). Zhao and Jing (2015) divided online interaction into two types: information interaction and interpersonal interaction. Bruhn et al. (2014) believe that in addition to the function of information transmission, online interaction also includes emotional interaction. It can be seen that scholars have sorted out the connotation and dimensions of online interaction from different perspectives, but few have done this for environmental improvement. Therefore, our study defines online interaction as the behavior involving information sharing and emotional exchange between consumers and short video publishers or other participants.

In the era of digital economy, social interaction among people has gradually shifted from offline to online, especially on new social platforms such as WeChat, Weibo, and TikTok. New social platforms serve as a tool for social communication, which are basically characterized by strong interactive relationship, high interactive autonomy and strong emotional resonance, and exert an important influence on people’s behavioral decision-making (Cao X. Y. et al., 2021). In the existing studies, the impact of online interaction on consumer behavior is mainly reflected in the promotion of consumer loyalty, participation intention and brand attachment (Luo et al., 2019; Choi and Kim, 2020; Xu X. et al., 2021). Shen et al. (2020) pointed out that online interaction is conducive to improving consumer value co-creation behavior with respect to the online tourism community. Zhang et al. (2022) found that customers share product experience during their interaction with others, which enhances their purchase intention. Consumers can easily and conveniently use short videos to engage in various forms of interaction, exchange of information with others and increase their knowledge, thus affecting their behavioral decisions (Zhao et al., 2022). Wu (2006) found that online interaction can lead to changes in consumers’ attitudes and further influence consumers’ online purchase intentions. According to Lin et al. (2022) social interaction enhances consumers’ environmentally responsible behavior by enhancing their perception of group norms and destination attachment. Wang et al. (2022b) found that online interaction can affect consumers’ emotions pertaining to the environment and subsequently their purchase intention for green products. Based on the above discussion, we believe that during the process of online interaction, consumers are likely to pay more attention to environmental issues by exchanging and sharing information related to the ecological environment, thus generating willingness to contribute to the environment and making them more willing to practice low-carbon tourism behaviors. Therefore, the following hypothesis was proposed:

*H3*: Online interaction has a significant positive impact on consumers’ low-carbon tourism behavioral intention.

### 2.5. The mediating role of empathy with nature

The concept of empathy was first proposed by Rogers (1959) and it refers to the ability of an individual to look into the inner world of others, which is specifically manifested as empathy for others’ circumstances. With the increasing global attention to the ecological environment, Tam (2013) further extends empathy with respect to relationship between people to the relationship between people and nature, and puts forward the concept of empathy with nature, which is defined as being able to perceive the situation of nature and understand it emotionally. It can be seen that empathy with nature changes people’s cognition and emotion towards it by establishing a connection between humans and nature, and facilitates the relationship between human and nature positively. Empathy with nature can also be understood as a combination of cognitive and emotional abilities of nature. Cognitive ability refers to the ability of individuals to distinguish and understand the situation of nature on the basis of past experience or through gaining environmental knowledge. Emotional ability refers to the ability of individuals to have emotional resonance with nature so that the aspects pertaining to deterioration of the ecological environment and exhaustion of natural resources can be acknowledged and understood (Wang L. T. et al., 2022). Individuals having strong empathy for nature are likely to pay more attention to the problems faced by nature and are able to think from the perspective of nature (Jing et al., 2022). Wang et al. (2022a) believed that empathy with nature should be taken as the starting point for the study of pro-environment behavior. Empathy with nature is helpful to enhance the emotional connection between humans and nature, and promote individuals’ environmentally responsible behavior. When empathy serves as a link between humans and nature, individuals are able to perceive the situation of nature and empathize with it considering how it is being harmed, which is likely to increase individuals’ willingness to take effective actions to solve environmental problems (Li et al., 2019).

The stimulus-organism-response (SOR) theory emphasizes that an individual’s internal emotional state can change on being stimulated by the external environment, thus generating specific behavioral responses (Mehrabian and Russell, 1974). This theory reveals that the mediating role of internal emotional state between external stimulus and behavioral response, has become an important analytical framework to explain people’s behavioral process, and has been applied to many fields, including retail (Lee and Yun, 2015), online consumer behavior (Xiao et al., 2019), and tourism field (Chen et al., 2020). The experiences (presence, perception of environmental education, online interaction) provided by the environment-friendly short videos can act as external environmental stimuli, thus affecting consumers’ internal emotional state (empathy with nature), and effectively stimulate consumers’ low-carbon tourism behavioral intention. Therefore, the SOR model provides a theoretical basis for revealing the intermediary transmission mechanism between environment-friendly short video experiences and consumers’ behavioral response (low-carbon tourism behavioral intention). In addition, the mediating role of empathy between external environmental stimuli and tourists’ environmentally responsible behavior has been supported (Li et al., 2019). Based on this, the following hypotheses were proposed:

*H4a*: Empathy with nature plays a mediating role between presence and consumers’ low-carbon tourism behavioral intention.

*H4b*: Empathy with nature plays a mediating role between perception of environmental education and consumers’ low-carbon tourism behavioral intention.

*H4c*: Empathy with nature plays a mediating role between online interaction and consumers’ low-carbon tourism behavioral intention.

### 2.6. The mediating role of perceived environmental responsibility

Mehrabian and Russell (1974) proposed the concept of perceived environmental responsibility from the stakeholders’ perspective, pointing out that perceived environmental responsibility is a sense of responsibility that individuals have when facing environmental problems, as well as the individual’s cognition of responsibility for maintaining the overall environment. This sense of responsibility can develop a more positive attitude in individuals towards environmental problems, and they believe that they have the ability to change the environmental status, and are more willing to make efforts for solving environmental problems (Yue et al., 2020). Ramkissoon et al. (2012) pointed out that tourists’ perceived environmental responsibility is a concrete embodiment of tourists’ fulfillment of their own responsibilities as stakeholders. As a key stakeholder of environmental management in destinations, tourists’ perceived environmental responsibility is an important driving force that promotes sustainable utilization of natural resources in tourism destinations. Existing studies have established models to analyze the formation mechanism of tourists’ environmental responsibility from different theoretical perspectives including the theory of planned behavior (Qiu et al., 2022), value-belief-norm theory (Sharma and Gupta, 2020), norm activation theory and social norm theory (Salinero et al., 2022). An individual’s sense of responsibility to adopt measures to solve specific environmental problems or prevent environmental quality deterioration, perceived environmental responsibility is an important antecedent variable for individuals to display environmental responsible behavior (Yue et al., 2020). A large number of studies have found a significant relationship between perceived environmental responsibility and green consumption and other pro-environmental behaviors (Luo et al., 2020; Yue et al., 2020).

The cognitive appraisal theory proposed by Lazarus (1991) explains the role of environment-friendly short video experiences on consumers’ low-carbon tourism behavioral intention. According to the theory, there is cognitive evaluation of the external stimulus and emotional response. Individuals’ cognitive evaluation of external stimulus is likely to trigger their emotional response, and prompt them to display coping behavior, thus demonstrating the “evaluation-emotion-response” path relationship (Bagozzi, 1992). This theory explains the intermediary transmission mechanism of environment-friendly short video experience on consumers’ low-carbon tourism behavioral intention. During the process of watching environment-friendly short videos, consumers are likely to evaluate their viewing experience (presence, perception of environmental education, online interaction), which is internalized as perceived environmental responsibility, so that consumers display a profound sense of responsibility for the ecological environment, and low-carbon tourism behavioral intention is generated, which facilitates realization of the path of “evaluation-emotion-response.” In addition, according to the norm activation theory (Schwartz, 1977), environment-friendly short video experience (including presence, perception of environmental education, and online interaction) can facilitate realization of the negative impact of non-environmental protection behavior on the environment in individuals, generate awareness regarding their responsibility for environmental deterioration and promote practice of low-carbon tourism behaviors. Accordingly, the following hypotheses were proposed:

*H5a*: Perceived environmental responsibility plays a mediating role between presence and consumers’ low-carbon tourism behavioral intention.

*H5b*: Perceived environmental responsibility plays a mediating role between the perception of environmental education and consumers’ low-carbon tourism behavioral intention.

*H5c*: Perceived environmental responsibility plays a mediating role between online interaction and consumers’ low-carbon tourism behavioral intention.

**Figure 1:**
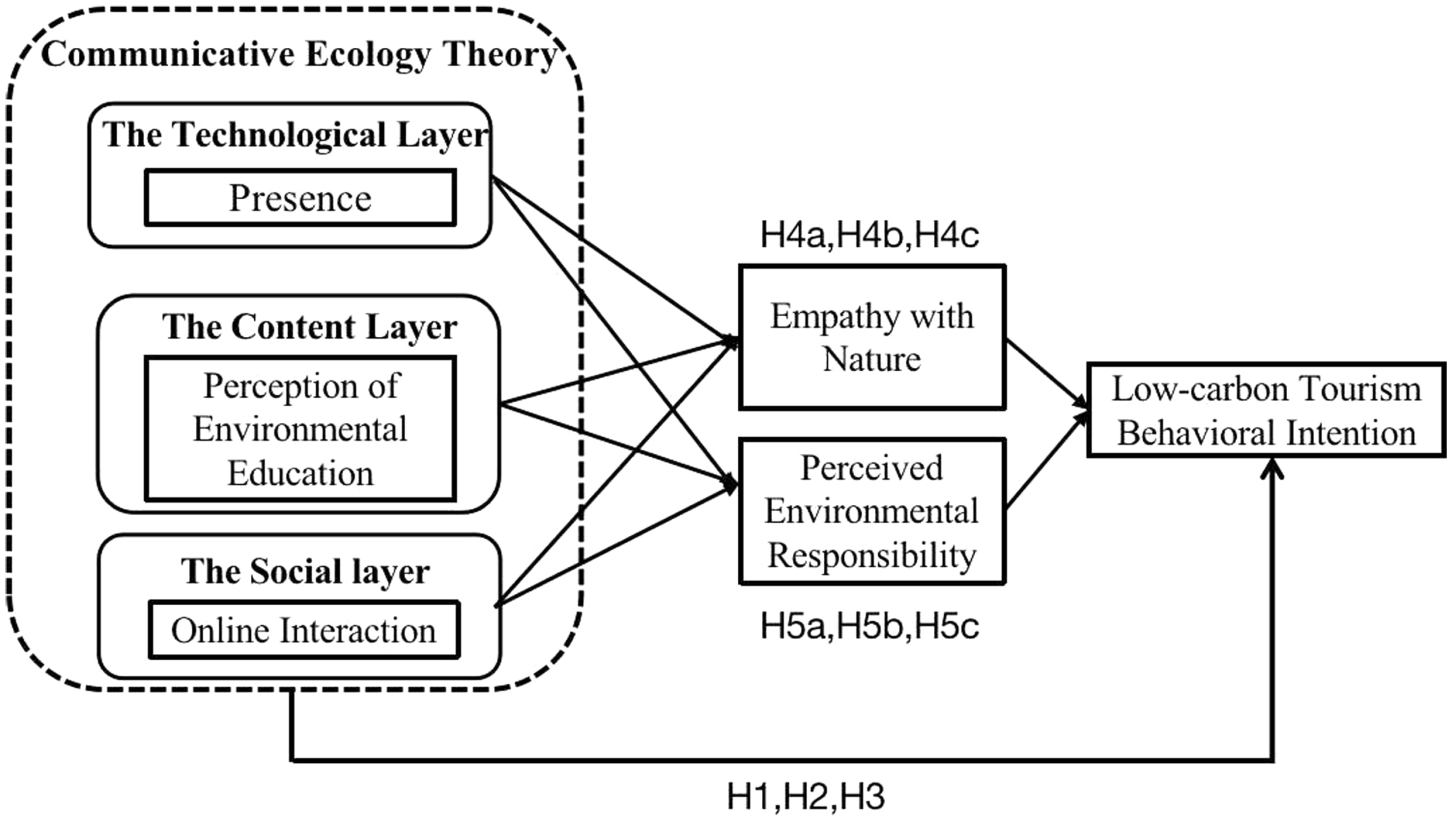
Theoretical model.

In light of literature reviews and practical investigations, our study establishes a meditation model based on empathy with nature and perceived environmental responsibility according to the theoretical framework of stimulus-organism-response. Environment-friendly short video experiences are taken as environmental stimuli. Empathy with nature and perceived environmental responsibility are regarded as organic elements to clarify the internally psychological perception of consumers. The consumers’ low-carbon tourism behavioral intention is considered as the final behavioral response. The theoretical model constructed in our study is illustrated in Figure 1.

## 3. Research design

### 3.1. Measurement of variables

All variables in our study were measured with the help of the existing scale. The English scale items referred to in our study were translated into Chinese by professional translators, and the accuracy of translation was ensured by the back translation method. Considering that our study explored the impact of environment-friendly short video experience on consumers’ low-carbon tourism behavioral intention, focus group interviews were conducted for further revision of the items. The focus group consisted of 7 interviewees, who can be categorized into three groups: (1) tourists having low-carbon tourism behavioral intention and those who have displayed low-carbon tourism behaviors after watching environment-friendly short videos; (2) tourists having low-carbon tourism behavioral intention but have not yet demonstrated low-carbon tourism behavior after watching environment-friendly short videos; (3) tourists in whom low-carbon tourism behavioral intention has not yet been generated even after watching environment-friendly short videos. On this basis, we conducted a preliminary survey on 90 individuals who had watched environment-friendly short videos. Based on the preliminary survey results, the content of the questionnaire was further revised and improved. Likert’s 7-point scale was used in all the scales, in which score 1 represented “completely disagree” and 7 represented “completely agree.” Specifically, the four items pertaining to presence were mainly adapted from the research of Xu X. Y. et al. (2021) and Kim et al. (2022). The items pertaining to perception of environmental education were mainly adapted from the scale developed by Yu et al. (2022) and Li et al. (2019), and contained four items. The three items pertaining to online interaction included in the scale, were mainly based on the research of Wu (2006) and Yoo and Lee (2010). The three items measuring empathy with nature were taken from the studies of Li et al. (2019) and Tam (2013). The three items measuring perceived environmental responsibility were mainly taken from the research of Punzo et al. (2019). The six items measuring low-carbon tourism behavioral intention were mainly adapted from the scale developed by Dai et al. (2022) and Zhang et al. (2017a).

### 3.2. Data collection and sample distribution

Since our study explored the impact of environment-friendly short video experience on consumers’ low-carbon tourism behavioral intention, the data was mainly collected through the online survey platform (“Sojump”). First, a group of respondents were randomly selected, and the data was collected *via* the questionnaire through the snowballing method. A screening mechanism was set up through the questionnaire for the selection of respondents, and only those having experience in watching environment-friendly short videos were allowed to respond. The questionnaire was officially circulated between August 3, 2022 and August 28, 2022. A total of 1,070 questionnaires were collected, 284 invalid questionnaires were excluded, and ultimately 786 valid questionnaires were obtained.

With respect to sample composition, 40.14% of the sample comprised males and 59.86 comprised females. The proportion of respondents aged 18 to 30 years was the highest (69.20%), followed by those aged 31 to 40 years (26.3%). The percentage of respondents having monthly income 2,000–6,000 CNY was the highest (63.6%). The proportion of undergraduate students was the highest (58.7%). Hence, on observation of the sample distribution it was found that it mostly comprised young participants, who are the main audience of short videos and have strong acceptance towards new technologies (Pan et al., 2016). Therefore, the sample of our study exhibited the characteristics of mainstream groups in the tourism consumption market in the age of digital media.

## 4. Data analysis and hypothesis testing

### 4.1. Normality test

The maximum likelihood method has been adopted to estimate the parameters of the research model. The sample data must obey a normal distribution, which is the preconditions for using this method. Through SPSS 27.0 software, it has been found that the absolute value of the skewness coefficient of all the items involved in our study ranges from 0.001 to 0.297 and less than 3, and that the absolute value of the kurtosis coefficient ranges from 0.311 to 1.695 and less than 10. Therefore, the sample data in our study obeys a normal distribution (Kline, 2011).

### 4.2. Common method variance test

In order to minimize the influence of common method variance, prior control measures such as anonymous filling and shuffling the order of questionnaire questions were adopted; Harman’s single-factor method was used to test common method variance existed. The results of exploratory factor analysis showed that the first factor before rotation explained 36.13% of the variation of all items, which was less than the critical value standard of 50%, indicating that the homologous method variation of data was well controlled in our study. In addition, confirmatory factor analysis of single and multi-factor models was performed to test the common method variance, and the results showed that there was a significant difference between the two models [Δx^2^ (15) = 4517.53, *p* < 0.001]. Again, it showed that research data were less likely to be affected by common method variance (Mcfarlin and Sweeney, 1992).

### 4.3. Reliability and validity test

Cronbach’s α value was used to determine whether the measurement tool was stable and reliable. The analysis results are illustrated in Table 1. The Cronbach’s α value of each variable ranged from 0.812 to 0.896, greater than 0.7, indicating good reliability of all variables in our study.

**Table 1:** Scale items and reliability test.

**Construct**	**Item**	**Cronbach’s α**
Presence	PRE1. When watching short videos about environmental protection, I feel like I am really ‘a part of the virtual scene.	0.882
	PRE2. When watching short videos about environmental protection, I feel that the things in the videos (animals, plants, etc.) are real.	
	PRE3. When watching short videos about environmental protection, I feel that things (animals, plants, etc.) in the videos are right in front of me.	
	PRE4: When watching short videos about environmental protection, I feel like I can touch the people or things in the videos.	
Perception of Environmental Education	PEE1. Short videos about environmental protection provide a rich description of ecological and environmental issues and ecosystem knowledge.	0.896
	PEE2. By watching short videos about environmental protection, I gain lot of ecological knowledge.	
	PEE3. After watching short videos about environmental protection, I realized the importance of protecting the environment.	
	PEE4. After watching short videos about environmental protection, I realized that human beings should respect and treat nature well.	
Online Interaction	OINT1. While watching short videos about environmental protection, I was able to interact with the publishers of the video.	0.812
	OINT2. While watching short videos about environmental protection, I can share and exchange information with others	
	OINT3. While watching short videos about environmental protection, I can make comments.	
Empathy with Nature	EWN1. I can perceive the damage done to the creatures in the natural environment in the video.	0.872
	EWN2. I can imagine the difficult situation of animals and plants in the video.	
	EWN3. I have great concern and sympathy for the animals and plants that were harmed in the video.	
Perceived Environmental Responsibility	PER1. Protecting the ecological environment is not only the responsibility of the government and environmental protection organizations, but also my responsibility.	0.883
	PER2. I will take the initiative to gain knowledge about environmental protection.	
	PER3. I should take the responsibility to protect the environment.	
Low-carbon Tourism Behavioral Intention	LTBI1. I would like to reduce the use of disposable items during travel.	0.863
	LTBI2. I take care to keep the destination clean and take away the garbage generated during the trip.	
	LTBI3. I will choose low-carbon transportation (such as public transport, bicycle, etc.) in the tourist destination.	
	LTBI4. I would choose a green and low-carbon hotel.	
	LTBI5. I would choose activities requiring lower energy consumption.	
	LTBI6. I will promote low-carbon tourism among others.	

Our study used confirmatory factor analysis to test the validity of the scale. The goodness of fit results showed that the fit indices of the measurement model reached the acceptable level (X^2^/DF = 4.230, RMSEA = 0.064, SRMR = 0.041, GFI = 0.885, IFI = 0.936, NFI = 0.917, TLI = 0.924, CFI = 0.935). The convergent validity analysis results of all variables are depicted in Table 2. The standardized factor loading is between 0.665 and 0.864, and the CR value is between 0.816 and 0.898, which are greater than the critical value 0.7. The AVE values are all greater than the critical value 0.5, indicating that all variables had good convergent validity (Fornell and Larcker, 1981).

**Table 2:** Confirmatory factor analysis.

Variable	Item	Significance of parameter	Convergent validity		
		Unstd.	S.E.	*z*	*p*	Std.	CR	AVE
Presence	PRE1	1.000				0.820	0.887	0.663
	PRE2	1.243	0.048	26.155	***	0.830		
	PRE3	1.017	0.038	26.432	***	0.837		
	PRE4	1.112	0.047	23.685	***	0.769		
Perception of Environmental Education	PEE1	1.000				0.838	0.898	0.687
	PEE2	1.083	0.039	27.502	***	0.834		
	PEE3	1.082	0.039	27.850	***	0.841		
	PEE4	0.845	0.032	26.085	***	0.803		
Online Interaction	OINT1	1.000				0.819	0.816	0.597
	OINT2	0.729	0.038	19.050	***	0.740		
	OINT3	0.858	0.044	19.292	***	0.756		
Empathy with Nature	EWN1	1.000				0.831	0.874	0.698
	EWN2	1.025	0.039	26.436	***	0.864		
	EWN3	1.028	0.041	24.952	***	0.810		
Perceived Environmental Responsibility	PER1	1.000				0.840	0.885	0.719
	PER2	1.034	0.038	27.281	***	0.844		
	PER3	0.938	0.034	27.848	***	0.860		
Low-carbon Tourism Behavioral Intention	LTBI1	1.000				0.672	0.865	0.517
	LTBI2	1.156	0.068	16.981	***	0.674			LTBI3	1.041	0.062	16.779	***	0.665		
LTBI4	1.403	0.075	18.785	***	0.756		
LTBI5	1.377	0.073	18.938	***	0.763		
LTBI6	1.556	0.081	18.189	***	0.775		

Std., standardized factor loading; Unstd., unstandardized factor loading; S.E., standard error; CR, composite reliability; AVE, average variance extracted.

*** *p* < 0.001.

In addition, our study tested the discriminant validity by comparing the square root value of AVE of all variables and the correlation coefficient between the variables. The results are illustrated in Table 3. The square root value of AVE of each variable is greater than the correlation coefficient between these variables. Therefore, all variables have acceptable discriminant validity.

**Table 3:** Discriminant validity test.

	PRE	PEE	OINT	EWN	PER	LTBI
PRE	0.814					
PEE	0.129	0.829				
OINT	0.230	0.283	0.773			
EWN	0.325	0.309	0.226	0.835		
PER	0.364	0.356	0.257	0.344	0.848	
LTBI	0.573	0.547	0.322	0.506	0.597	0.719

The off-diagonal items are correlation coefficients; on-diagonal items are square roots of AVE; PRE, presence; PEE, perception of environmental education; OINT, online interaction; EWN, empathy with nature; PER, perceived environmental responsibility; LTBI, low-carbon tourism behavioral intention.

### 4.4. Hypotheses testing

#### 4.4.1. Main effect test

**Figure 2:**
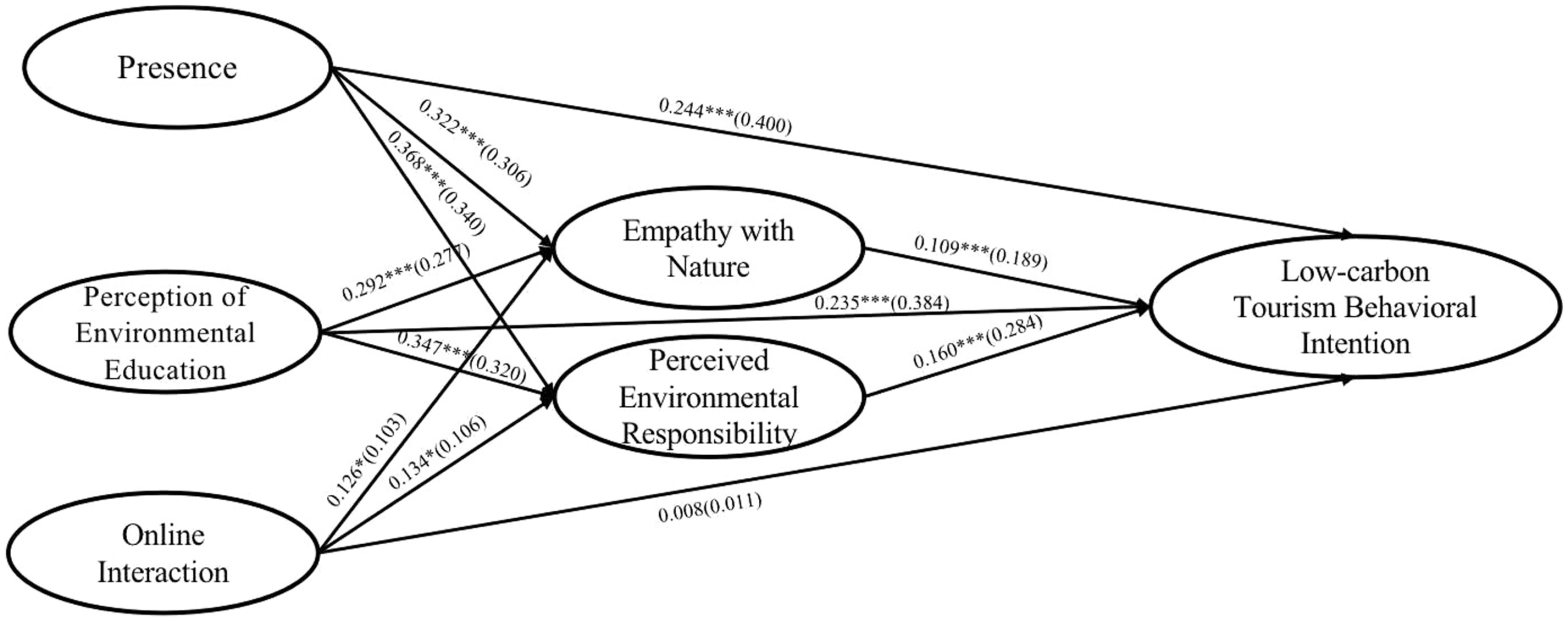
The path analysis. Unstandardized estimation (standardized estimation); ***p < 0.001;**p < 0.01; and *p < 0.05.

In our study, AMOS 26.0 software was used to estimate the parameters of the research model by using maximum likelihood method, and the structural model fitted well (X2/DF = 4.285, RMSEA = 0.065, SRMR = 0.045, GFI = 0.884, IFI = 0.934, NFI = 0.916, SRMR = 0.045, GFI = 0.884, IFI = 0.934, NFI = 0.916, TLI = 0.923, CFI = 0.934). The results of path analysis are shown in Figure 2, the presence (β = 0.244, *p* < 0.001, BC 95%CI = [0.194, 0.295]), and the perception of environmental education (β = 0.235, *p* < 0.001, BC 95%CI = [0.192, 0.283]) has a significant positive impact on consumers’ low-carbon tourism behavioral intention. Therefore, H1 and H2 were supported. However, the effect of online interaction on consumers’ low-carbon tourism behavioral intention was not significant (β = 0.008, *p* > 0.05, BC 95%CI = [−0.034, 0.050]), so hypothesis H3 was not supported. In addition, the presence (β = 0.322, *p* < 0.001, BC 95%CI = [0.241, 0.412]), the perception of environmental education (β = 0.292, *p* < 0.001, BC 95%CI = [0.205, 0.379]) and online interaction (β = 0.126, *p* < 0.05, BC 95%CI = [0.014, 0.242]) had a positive effect on empathy with nature. Empathy with nature can further motivate consumers’ low-carbon tourism behavioral intention (β = 0.109, *p* < 0.001, BC 95%CI = [0.076, 0.144]), suggesting that the variable may have mediating effects between presence, perception of environmental education, online interaction and consumers’ low-carbon tourism behavioral intention. Meanwhile, presence (β = 0.368, *p* < 0.001, BC 95%CI = [0.287, 0.459]), perception of environmental education (β = 0.347, *p* < 0.001, BC 95%CI = [0.267, 0.433]) and online interaction (β = 0.134, *p* < 0.05, BC 95%CI = [0.038, 0.236]) had a positive promoting effect on perceived environmental responsibility. Perceived environmental responsibility had a significant positive effect on consumers’ intention to indulge in low-carbon tourism behavior (β = 0.160, *p* < 0.001, BC 95%CI = [0.124, 0.201]), indicating that there may be a mediating effect between presence, perception of environmental education, online interaction and consumers’ low-carbon tourism behavioral intention.

#### 4.4.2. The mediating effect test

Following the suggestion of Preacher et al. (2007), our study used the bootstrap method to test the mediating effect of empathy with nature and perceived environmental responsibility. The confidence level was set at 95% and the number of iterations was set at 5,000. The test results are shown in Table 4, empathy with nature has significant mediating effect between presence (β = 0.035, *p* < 0.001, BC 95%CI = [0.023, 0.051]), perception of environmental education (β = 0.032, *p* < 0.001, BC 95%CI = [0.021, 0.048]), online interaction (β = 0.014, *p* < 0.05, BC 95%CI = [0.002, 0.029]) and consumers’ low-carbon tourism behavioral intention. Therefore, hypotheses H4a, H4b and H4c are further supported. In addition, perceived environmental responsibility played a significant mediating effect between presence (β = 0.059, *p* < 0.001, BC 95%CI = [0.042, 0.081]), perception of environmental education (β = 0.056, *p* < 0.001, BC 95%CI = [0.040, 0.078]), online interaction (β = 0.021, *p* < 0.01, BC 95%CI = [0.007, 0.039]), and consumers’ low-carbon tourism behavioral intention. Therefore, the hypotheses H5a, H5b and H5c are further supported. According to the direct effects of presence (β = 0.244, *p* < 0.001) and perception of environmental education (β = 0.235, *p* < 0.001) on consumers’ low-carbon tourism behavioral intention, it can be seen that empathy with nature and perceived environmental responsibility play a partial mediating role between presence, perception of environmental education and consumers’ low-carbon tourism behavioral intention. The direct effect of online interaction on consumers’ low-carbon travel behavior intention (β = 0.008, *p* > 0.05) was not significant, indicating that empathy with nature and perceived environmental responsibility played a mediating role between online interaction and consumers’ low-carbon tourism behavioral intention.

**Table 4:** Indirect and direct effects.

Paths	Estimates	Product of coefficients		Bootstrapping	
				Bias-Corrected 95% CI	
		S.E.	*z*	Lower limit	Upper limit
Indirect effects					
PRE --> EWN-->LTBI	0.035	0.007	5.000	0.023	0.051
PEE --> EWN-->LTBI	0.032	0.007	4.571	0.021	0.048
OINT --> EWN-->LTBI	0.014	0.007	2.000	0.002	0.029
PRE --> PER-->LTBI	0.059	0.010	5.900	0.042	0.081
PEE --> PER-->LTBI	0.056	0.010	5.600	0.040	0.078
OINT --> PER-->LTBI	0.021	0.008	2.625	0.007	0.039
Direct effects					
PRE --> LTBI	0.244	0.022	11.261	0.194	0.295
PEE --> LTBI	0.235	0.021	11.232	0.192	0.283
OINT --> LTBI	0.008	0.020	0.370	−0.034	0.050

S.E., standard error; PRE: presence; PEE, perception of environmental education; OINT, online interaction; EWN, empathy with nature; PER, perceived environmental responsibility; LTBI, low-carbon tourism behavioral intention.

## 5. Conclusion and discussion

### 5.1. Conclusion

Based on the analytical framework of CET and SOR theory, our study focuses on environment-friendly short videos, constructs a mediation model based on empathy with nature and perceived environmental responsibility, and empirically tests the relationship between short video experience (presence, perception of environmental education, online interaction) and consumers’ low-carbon tourism behavioral intention. The results are as follows:

First, the stronger the presence and perception of environmental education consumers experience when watching environment-friendly short videos, the more inclined they are to indulge in low-carbon tourism behavior. Second, the presence, perception of environmental education and online interaction offer positive stimulation to consumers through the environment-friendly short videos, which can make consumers empathize with nature and their low-carbon tourism behavioral intention is enhanced. Thirdly, perceived environmental responsibility plays a mediating role between presence, perception of environmental education, online interaction and consumers’ low-carbon tourism behavioral intention. In other words, presence, perception of environmental education and online interaction can positively impact consumers’ low-carbon tourism behavioral intention indirectly due to the perceived environmental responsibility.

### 5.2. Discussion

It is found that presence and perception of environmental education have a positive promoting effect on consumers’ low-carbon tourism behavioral intention. Existing studies have shown that presence, as a kind of immersive feeling in the virtual environment, is a key predictor of consumers’ behavioral intention (Yung et al., 2021). At the same time, existing studies on perception of environmental education and tourists’ behavioral intention are mostly carried out based on field tourism scenarios (Li et al., 2019), and little attention is paid to the relationship between ecological environment knowledge conveyed by short videos and tourists’ environmental responsibility behavioral intention. Therefore, the research findings not only deepen the academic community’s understanding of the antecedent variables of consumers’ low-carbon tourism behavioral intention, but also verify the conclusion of Yang and Zhang (2020) on the relationship between new media persuasion and audience behavior from the perspective of CET. However, the positive impact of online interaction on consumers’ low-carbon tourism behavioral intention has not been supported, which is inconsistent with the research conclusion of Wang et al. (2022b). One possible explanation is that most previous studies explored the impact of online interaction on consumers’ behavioral intention based on online live shopping scenarios. Since shopping scenarios involve consumers’ purchasing decisions, the degree of online interaction is higher. However, the main purpose of our study was to explore the communication effect of environment-friendly short videos, which does not involve the consumers’ purchase decision-making process and has a low interaction frequency, so it is difficult to stimulate consumers’ low-carbon tourism behavioral intention.

The results of our study show that empathy with nature plays a mediating role between presence, perception of environmental education, online interaction and consumers’ low-carbon tourism behavioral intention. Therefore, empathy with nature provides an effective way to understand the effect mechanism of propagation of environment-friendly short videos. Previous studies have found that empathy with nature gets stimulated in the tourists during the process of travel, and generates intention to indulge in environmentally responsible behavior (Li et al., 2019; Wang L.T. et al., 2022). In the context of new media communication, empathy with nature stimulated by environment-friendly short videos can also promote consumers’ willingness to display low-carbon tourism behavior, which provides meaningful insights pertaining to empathy with nature.

Although studies in the field of tourism have revealed the positive effect of perceived environmental responsibility on pro-environment behavior (Ramkissoon et al., 2012; Yue et al., 2020), they have not focused on its role in the relationship between environment-friendly short videos and consumers’ intention to conduct low-carbon tourism behavior. The findings of the present study revealed that perceived environmental responsibility plays an important mediating role in the relationship between presence, perception of environmental education, online interaction and consumers’ low-carbon tourism behavioral intention.

### 5.3. Theoretical contributions

The theoretical contributions of our study mainly pertain to three aspects. First of all, current studies on the application of short videos in the field of tourism are mainly limited to the formulation of destination marketing strategies (Ke et al., 2022; Zhao et al., 2022), and little attention is paid to the influencing mechanism between short video and consumers’ behavioral intentions. Our study extends the influencing effect of short video to the level of consumers’ low-carbon tourism behavioral intention, and thus contributes to expansion of research on short video and low-carbon tourism. In addition, the existing studies have mostly explored the motivation of tourists to act in an environmentally responsible manner on the basis of the theory of planned behavior and norm activation theory (Qiu et al., 2022; Salinero et al., 2022). Our study focuses on the CET to deeply analyze the influencing mechanism of environment-friendly short videos on consumers’ low-carbon tourism behavioral intention, and enriches the related research on the influencing effect of short videos and antecedent variables of consumers’ behavioral intention with regard to low-carbon tourism (Zhang et al., 2017b; Fakfare and Wattanacharoensil, 2022; Ke et al., 2022; Zhao et al., 2022).

Secondly, theoretical contributions of empirical studies include theory construction and theory testing, and exploring new mediating and moderating variables in the existing theoretical relationships is an important part of theory construction (Colquitt and Zapata-Phelan, 2007). Our study tries to open the “black box” between short videos and consumers’ intention to low-carbon tourism behavior and extend the driving process of consumers’ intention to low-carbon tourism behavior from cognitive motivation to emotional motivation, which not only provides a unique explanatory perspective for the formation mechanism of consumers’ intention for low-carbon tourism behavior. It also provides a new way for the academicians to explore the relationship between short videos and consumers’ environmentally responsible behavior from the perspective of empathy with nature and perceived environmental responsibility.

In addition, our study introduces CET from the field of communication to the field of low-carbon tourism, thus expanding the boundary of its application. The CET originated from the field of communication studies, focuses on the effects of media communication (Altheide, 1994; Foth and Hearn, 2007), and has been applied to the field of consumer behavior in recent years. For example, some scholars have discussed the driving mechanism of users’ willingness to continue using pages of SNS based on the CET (Seol et al., 2016). As a new media, the communication effect of short video is also applicable to the CET. Therefore, our study attempts to introduce the CET in exploring the mechanism of environment-friendly short video experience on consumers’ behavioral intention of low-carbon tourism from the perspective of empathy with nature and perceived environmental responsibility, which not only promotes the development but also the application of CET in the field of low-carbon tourism. Moreover, the three layers of CET are embodied as presence (technological layer), perception of environmental education (content layer) and online interaction (social layer), which enrich and expand the connotation of CET.

### 5.4. Practical implications

First of all, our study provided evidence that watching environment-friendly short video (presence, perception of environmental education, online interaction) directly or indirectly exerts a positive impact on consumers’ low-carbon tourism behavioral intention, indicating that creating high-quality environment-friendly short videos is a fruitful way to motivate individuals to practice low-carbon tourism behavior. With respect to the government and tourist destinations, the findings of the research are likely to provide theoretical guidance for promoting the concept of low-carbon tourism through new media communication channels. The government and the tourist destinations should not only improve their ability to make environment-friendly short videos, but also encourage and guide the public to shoot and share high-quality short videos on the theme “environmental protection,” so as to enhance public’s awareness of low-carbon environmental protection and to arouse intention of low-carbon tourism behavior. Specifically, in the production of short videos, artificial intelligence (AI), virtual reality (VR), and augmented reality (AR) technology should be used to design a more vivid video content, so that the audience experiences a sense of immersiveness. For example, a more realistic ecological destruction scene can be presented to the users, and the content related to environmental degradation can be clearly presented in a highly three-dimensional visual format, so as to improve their level of immersive experience and spatial presence, and thus stimulate their low-carbon tourism behavioral intention. Secondly, we should pay attention to the in-depth mining of the theme and content that imparts environmental knowledge, and integrates it into the short videos from multiple perspectives. For example, by assessing the cognitive ability and preferences of the audience, developing different theme pertaining to environmental knowledge and content for precision marketing, to enhance the effect of environmental education; guide users to share their experience and knowledge of environmental protection, and effectively manage and monitor user-generated content to eliminate unhealthy content; create a relaxed and pleasant experiential learning environment, let the users accept the edifying of environmental knowledge through melodramatic performance, story explanation, knowledge competition, game interaction and other ways, and truly realize the educational function of environment-friendly short videos. In addition, the level of interaction in short videos should be improved to provide a congenial environment for the users to enhance their communication and interaction. For example, by encouraging users to actively search, comment and share information, and make short videos a contact channel for sharing knowledge about environmental protection; continuous improvement of the platform interface to ensure smooth real-time interaction between users; ensure real-time multi-channel interaction between users and media; provide simple and easy-to-use tools to meet the needs of users for communication anytime and anywhere, and achieve positive interaction between users, tourism destinations and the government.

Secondly, the results of our study show that, as a positive external stimulus, environment-friendly short videos can make users empathize with nature, and thus stimulate their behavioral intention of low-carbon tourism. It can be seen that empathy with nature plays an important role in promoting low-carbon tourism behavior. AI technology and social media can be used to publicize the importance of protection of natural environment and ecological habitat *via* multiple channels and wide areas by the tourism destinations and the governments. VR, AR and other technologies should be used to vividly present those animals and plants that have lost their habitats or even suffered extinction due to the destruction of the ecological environment in an anthropomorphic way, so as to enhance tourists’ empathy for the natural ecology, and their attitude towards the protection of the destination environment. In addition, abstract concepts such as “natural environment,” “ecological destruction,” and “environmental knowledge” can be concretized through anthropomorphic means. For example, the natural environment can be anthropomorphized with the help of stories and stage performances to help improve tourists emotional connection with the natural environment. In addition, the destination can set a series of environment-friendly signs, to draw tourists’ attention towards the destination environment and resources through visual stimulation of the sign system, encourage tourists to empathize with nature, and practice low-carbon tourism behavior.

Finally, the results revealed that perceived environmental responsibility plays a mediating role between environment-friendly short videos and consumers’ intention to conduct low-carbon tourism behavior, indicating that perceived environmental responsibility is an important factor that affects consumers’ low-carbon tourism behavioral intention. The government should strengthen the publicity and provide education on environmental issues, encourage the public to indulge in practices pertaining to environmental protection, and cultivate a sense of environmental responsibility in the public through various channels. For example, social media platforms such as TikTok, WeChat, and Weibo can be used to impart environmental knowledge, generate awareness regarding the current situation of environmental problems such as air pollution and ecological damage of different kinds, so that the public is able to realize the seriousness of environmental protection and becomes more aware of their responsibility for environmental problems. In addition, the government and the relevant departments should actively guide the public to participate in environmental protection practices and encourage them to participate in environmental co-governance through various means, so as to enhance their awareness of their environmental responsibility. For the tourism destination, the motivation of tourists’ low-carbon tourism behavior is closely related to the tourism atmosphere provided and created by the destination. Therefore, while promoting green products such as green food, low-carbon hotels, low-carbon transportation, etc., environmental knowledge and information pertaining to the benefits of the green products to the environment can be provided to them in the tourist destinations, so as to reduce the perceived risks caused by insufficient knowledge of the green products. At the same time, the destination should reduce the cost of green consumption or provide preferential programs for green consumption, enrich the channels and ways of buying green products, so as to encourage tourists to change the traditional consumption mode and enhance their willingness towards low-carbon tourism behavior. The individual consumers should be aware of the importance of their own behavior to environmental protection and the sense of responsibility as social citizens to protect the environment. They should pay attention to the environmental issues, and actively participate in environmental protection related activities through various means, gradually cultivate environmental responsibility, and indulge in low-carbon tourism behavior.

### 5.5. Research limitations and future prospects

First of all, due to the limitations of cross-sectional data, the potential threat caused by common method variance could not be eliminated. Therefore, it is necessary for future studies to not only optimize the study design, but also employ the longitudinal tracking method for measurement, so as to reflect the logical relationship between variables more accurately. Secondly, our study only explores the relationship between environment-friendly short video experience and consumers’ low-carbon tourism behavioral intention. Whether transmission of information pertaining to environmental protection *via* pictures and text has the same effect as short videos, needs to be empirically tested in subsequent studies. In addition, our study mainly investigates the impact of environment-friendly short video experience (presence, perception of environmental education, online interaction), empathy with nature, perceived environmental responsibility on consumers’ low-carbon tourism behavioral intention. However, in real life, there are other factors that affect the consumers’ low-carbon tourism behavioral intention, such as environmental values, environmental concern, self-efficacy, etc. Future research can explore the effect of such factors on consumers’ behavioral intention of low-carbon tourism.

## Data availability statement

The original contributions presented in the study are included in the article/supplementary material, further inquiries can be directed to the corresponding author.

## Author contributions

XC: conceptualization, investigation, resources, software, writing—original draft, and writing—review and editing. Z-fC: data curation, funding acquisition, project administration, supervision, and visualization. XC and Z-fC: formal analysis, methodology, and validation. All authors contributed to the article and approved the submitted version.

## Funding

This research was supported by the following agencies: Humanities and Social Science Foundation of Ministry of Education of China (grant number: 22YJC860003); 2022 project of Guangzhou Philosophy and Social Sciences Planning (grant number: 2022GZGJ80).

## Conflict of interest

The authors declare that the research was conducted in the absence of any commercial or financial relationships that could be construed as a potential conflict of interest.

## Publisher’s note

All claims expressed in this article are solely those of the authors and do not necessarily represent those of their affiliated organizations, or those of the publisher, the editors and the reviewers. Any product that may be evaluated in this article, or claim that may be made by its manufacturer, is not guaranteed or endorsed by the publisher.
